# Effect of timing of bronchodilator therapy initiation on exacerbations in patients with chronic obstructive pulmonary disease: a retrospective cohort study

**DOI:** 10.1186/s12931-022-02184-6

**Published:** 2022-09-19

**Authors:** Hideyasu Yamada, Isao Matsumoto, Naoyuki Makita, Yoshifumi Arita, Nobuya Hayashi, Kurena Mitsuoka, Naoki Tashiro, Nobuyuki Hizawa

**Affiliations:** 1 Department of Pulmonary Medicine, University of Tsukuba, 1-1-1 Tennodai, Tsukuba, Ibaraki 305-8575 Japan; 2 AstraZeneca K.K., Osaka, Japan

**Keywords:** Annual exacerbation rate, Bronchodilator, COPD, Exacerbation, Therapy initiation

## Abstract

**Background:**

The benefit of prompt vs delayed treatment initiation with inhaled long-acting bronchodilators in reducing exacerbations in chronic obstructive pulmonary disease (COPD) is unclear. This study aimed to investigate if long-acting bronchodilator therapy initiation within 30 days of COPD diagnosis reduces exacerbation risk in patients with COPD.

**Methods:**

This was a retrospective cohort study of patients with COPD based on claims and electronic medical records data extracted from the Real World Data database. The index date (day 0) was the date of the first confirmed inpatient or outpatient COPD diagnosis between January 1, 2005, and December 31, 2018. Patients with COPD without an asthma diagnosis and aged ≥ 40 years at the index date were included. Patients who initiated inhaled long-acting bronchodilator therapy within the first 30 days (day 0 to day 29) were categorized into the “prompt therapy” group and the rest into the “delayed therapy” group. Time from day 30 post-diagnosis to the first exacerbation and annual exacerbation rate (AER) were evaluated for the overall population and those stratified by COPD phenotype, including chronic bronchitis (CB) and emphysema.

**Results:**

Compared with the delayed therapy group (n = 1516), time to first exacerbation was prolonged (hazard ratio 0.78; 95% confidence interval [CI] [0.70, 0.87]) and annual rates of moderate or severe exacerbations were lower (rate ratio 0.74; 95% CI [0.65, 0.84]) in the prompt therapy group (n = 1466). Similarly, time to first exacerbation was prolonged and AERs were lower in the prompt therapy group in the subgroups of patients with CB or emphysema.

**Conclusions:**

This is the first study to demonstrate a prolonged time to first exacerbation upon initiation of long-acting bronchodilators within 30 days of COPD diagnosis. A beneficial effect was also observed in patients with CB and emphysema. Our data support advising patients to initiate long-acting bronchodilators soon after COPD diagnosis.

**Supplementary Information:**

The online version contains supplementary material available at 10.1186/s12931-022-02184-6.

## Background

Chronic obstructive pulmonary disease (COPD) exacerbations are associated with an accelerated decline of lung function [1, 2], resulting in poor quality of life and poor prognosis [1, 3, 4]. An increase in the financial burden of COPD on healthcare systems has also been attributed to exacerbations [5]. Exacerbations are known to become more frequent as COPD progresses [6, 7], with the most important predictor of frequent exacerbations being a history of prior exacerbations [8].

COPD is classified into chronic bronchitis (CB) and emphysema [9]. An increase in bronchial wall thickness has been shown to be associated with an increase in the annual exacerbation rate (AER), suggesting a correlation between the CB phenotype and exacerbations [10]. In the Japanese population with COPD, the emphysema phenotype is more prevalent than the CB phenotype [11] and is similarly associated with exacerbations and poor prognosis [12]. Despite a large number of patients suspected of having COPD in Japan, treatment remains suboptimal. According to a previous survey of patients with suspected COPD with CB or emphysema aged > 45 years with a smoking history in Japan, 52% of participants were not diagnosed with COPD and < 16% of those diagnosed received an inhaled bronchodilator, which is the recommended first-line treatment according to the COPD guidelines of the Japanese Respiratory Society [13].

Data from placebo-controlled trials suggest that initiation of maintenance therapy with long-acting bronchodilators in the early stages of the disease, or in younger patients, may substantially improve COPD-related parameters, such as lung function, health-related quality of life, and exacerbation, and retard disease progression [14–18]. However, the association between early initiation of long-acting bronchodilator therapy after COPD diagnosis and COPD outcomes compared with delayed initiation remains to be elucidated. Therefore, understanding the clinical importance of early initiation of long-acting bronchodilator therapy is essential to promote prompt therapy initiation for patients with COPD. This study aimed to explore the impact of treatment initiation with long-acting bronchodilator therapy within 30 days of COPD diagnosis on exacerbation risk in patients with COPD.

## Methods

### Study design and study population

**Fig. 1:**
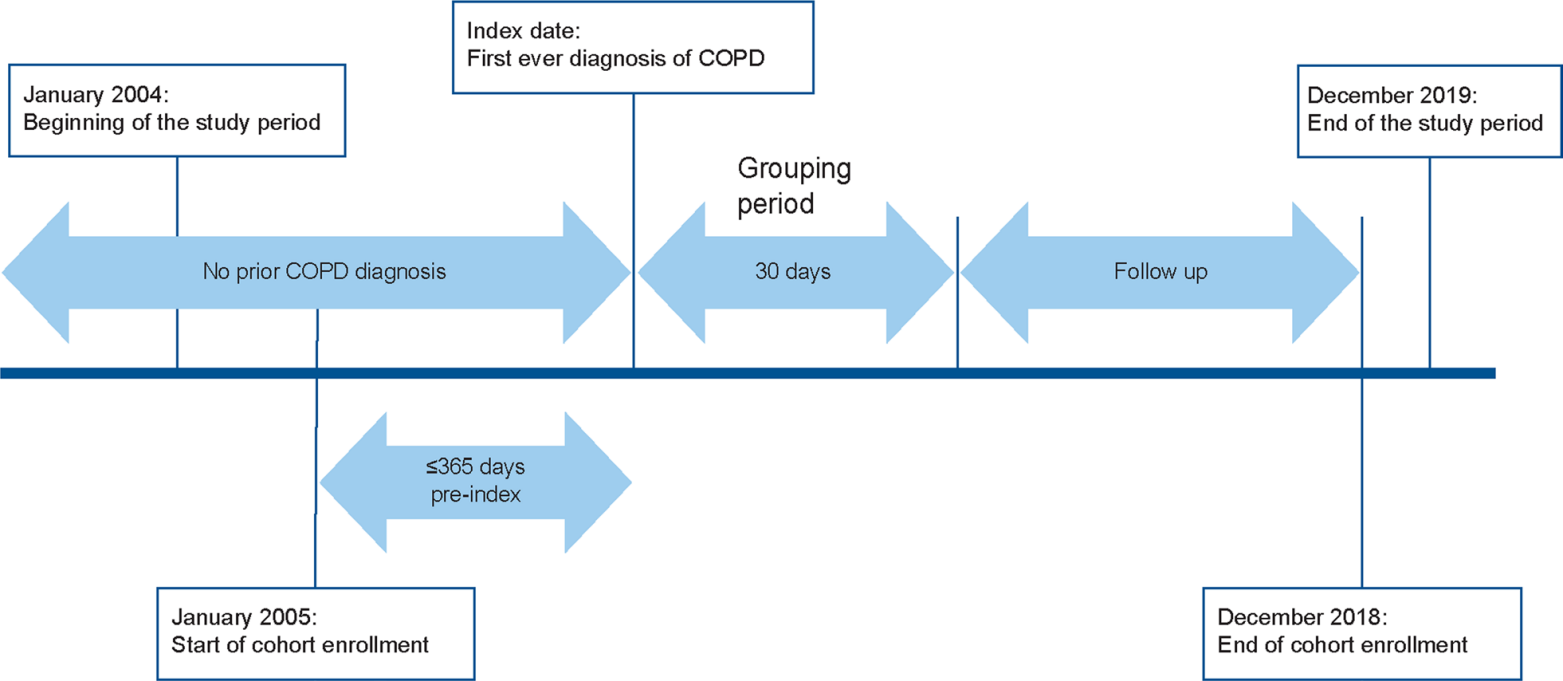
Study design. *COPD* chronic obstructive pulmonary disease

This was a retrospective cohort study of patients with COPD based on data extracted from a medical record–based database (Real World Data [RWD] database, Real World Data, Co., Ltd., Kyoto, Japan). The study period was from January 1, 2004, to December 31, 2019, and the cohort enrollment period was from January 1, 2005, to December 31, 2018, which was 1 year before the end of data availability, to allow accrual of sufficient follow-up for the study outcomes to be assessed. The index date (day 0) was the date of the first inpatient or outpatient COPD diagnosis (excluding suspected COPD) between January 1, 2005, and December 31, 2018, in the electronic medical record (EMR; Fig. 1).

The study protocol was approved by the NPO-MINS Institutional Review Board (Approval No. MINS-REC-210216) in 2021. The study was conducted in accordance with the Ethical Guidelines for Biomedical Research Involving Human Subjects, the ethical principles of the Declaration of Helsinki, and all relevant regulations applicable to noninterventional studies. The requirement for informed consent was waived because the available data in the RWD database were standardized and anonymized.

The study population consisted of incident patients with COPD without a prior diagnosis of COPD identified during the study period. The full analysis set included all incident patients with COPD at the index date, excluding those with an exacerbation during the grouping periods (periods of treatment initiation). The inclusion criteria were as follows: a first-time COPD diagnosis in the inpatient or outpatient setting in the cohort enrollment period, identified by a relevant diagnosis code (International Statistical Classification of Diseases and Related Health Problems, 10th Revision [ICD-10] codes J42, J43, and J44; any position; Additional file 1: Table S1) in the EMR; availability for follow-up 365 days before and after the index date; age ≥ 40 years at the index date; and at least one dispensation of a long-acting muscarinic antagonist (LAMA; R03K2), long-acting β_2_-agonist (LABA; R03A3), short-acting β_2_-agonist (R03A4), short-acting muscarinic antagonist (R03K1), LAMA + LABA (R03L2), inhaled corticosteroid (ICS) + LAMA, ICS + LABA (R03F1), or ICS + LAMA + LABA (R03L3) after the index date (Anatomical Therapeutic Chemical Classification codes listed in Additional file 1: Table S2). The exclusion criteria included a diagnosis of asthma in the inpatient or outpatient setting (ICD-10 codes J45 and J46; any position) during the study period or reversal of COPD diagnosis within 3 months.

Incident patients with COPD meeting the eligibility criteria were categorized into the “prompt therapy” group (treatment initiation with long-acting bronchodilators [LAMA, LABA, LAMA + LABA, ICS + LABA, or ICS + LAMA + LABA] within the 30-day post-diagnosis period [day 0 to day 29]) or the “delayed therapy” group (no treatment initiation with long-acting bronchodilators within the first 30 days).

### Outcomes

The primary outcome was the occurrence of an exacerbation after the grouping period. The time from the day following the grouping period to the first exacerbation and the AER of incident COPD were compared between the prompt and delayed therapy groups. Exacerbations were classified as moderate or severe. A moderate exacerbation was defined as an outpatient dispensation claim for a systemic corticosteroid (H02) at the time of COPD diagnosis in the EMR or an outpatient dispensation claim for an antibiotic (J01) commonly used to treat exacerbations at the time of upper or lower respiratory tract infection diagnosis (J00-J22) in the EMR. A severe exacerbation was defined as an inpatient admission record with a diagnosis code (any position) for a COPD exacerbation (J10, J11, J13, J14, J15, J16, J18, J20, J21, J22, J440, J441, J680, J851, C1, A481, B012, B052, or B250) and a dispensation claim for a systemic corticosteroid on the same day or within 7 days of the admission date in the EMR; a receipt of hospitalization from the Diagnosis Procedure Combination system with a diagnosis code (both primary and other) for COPD (J42, J43, or J44) as the causative disease and/or main disease for hospitalization; or an inpatient admission record with a diagnosis code (primary position) for acute respiratory failure (J80, J96, or R09.2) concomitant with a dispensation claim for a systemic corticosteroid on the same day or within 7 days of the admission date but no diagnosis with heart failure (I50), pneumothorax (J93), or myocardial infarction (I21 or I22) recorded during the same admission in the EMR. For moderate exacerbations, the date of medication dispensation was defined as the exacerbation date. For severe exacerbations, the date of admission was defined as the exacerbation date. If more than two exacerbations of any severity were identified within a 14-day period, the events were counted as the same event and the exacerbation date was recorded as the date of the first exacerbation. If the severity of these exacerbations differed, the exacerbation was classified as severe and the exacerbation date was recorded as the date of the earliest of all the events that occurred within the same 14-day window.

### Statistical analysis

Patient demographics and baseline characteristics are described using mean, standard deviation, and median for continuous variables, and number and percentage for categorical variables. The proportion of missing data is reported for each variable measured in the study.

Kaplan–Meier plots for the time to first exacerbation among incident patients with COPD are presented by treatment initiation groups (prompt therapy group and delayed therapy group). The proportion of events and censoring as well as the median time to first exacerbation were based on the Kaplan–Meier method, and hazard ratios (HRs) and corresponding 95% confidence intervals (CIs) between groups were calculated using a Cox proportional hazards model including the treatment initiation groups. The AER was analyzed based on a negative binomial model including the treatment initiation group with the logarithmic length of the follow-up period as an offset variable. The rate ratio (RR) and 95% CIs between groups were estimated based on the negative binomial model. Adjusted HRs and RRs and corresponding 95% CIs between groups were also estimated including the treatment initiation group and covariates in the pre-index period (age, sex, number of comorbidities, systemic corticosteroid use, antibiotic use, and inpatient with a COPD exacerbation or acute respiratory failure). Kaplan–Meier plots for the time to first exacerbation are presented by treatment initiation groups and by COPD phenotypes (CB [J42], emphysema [J43], and unclassified [J44]). CB or emphysema-type COPD was identified based on the corresponding diagnosis codes assigned from January 1, 2004, to December 31, 2018 (inclusive). The unclassified COPD phenotype included patients assigned the diagnosis code J44, including the first diagnosis from January 1, 2004, to December 31, 2018 (inclusive). The time to first exacerbation between the prompt therapy and delayed therapy groups was further compared in the following subgroups: (1) index date after November 1, 2013 (the time of LAMA + LABA therapy launch in Japan), and (2) index date after April 1, 2018 (the time when COPD guidelines began recommending LAMA + LABA use for COPD in Japan). Kaplan–Meier plots for the time to first exacerbation are also presented by subgroups restricted to patients with an incident pure COPD diagnosis and by subgroups stratified by post-diagnosis treatment initiation time points as follows: day 0 (i.e., the day of diagnosis or the index date) vs day 1 and beyond; from day 0 to day 59 (i.e., within 60 days of the index date) vs day 60 and beyond (i.e., 60 days after the index date); and from day 0 to day 89 (i.e., within 90 days of the index date) vs day 90 and beyond (i.e., 90 days after the index date).

## Results

### Patients characteristics

**Fig. 2:**
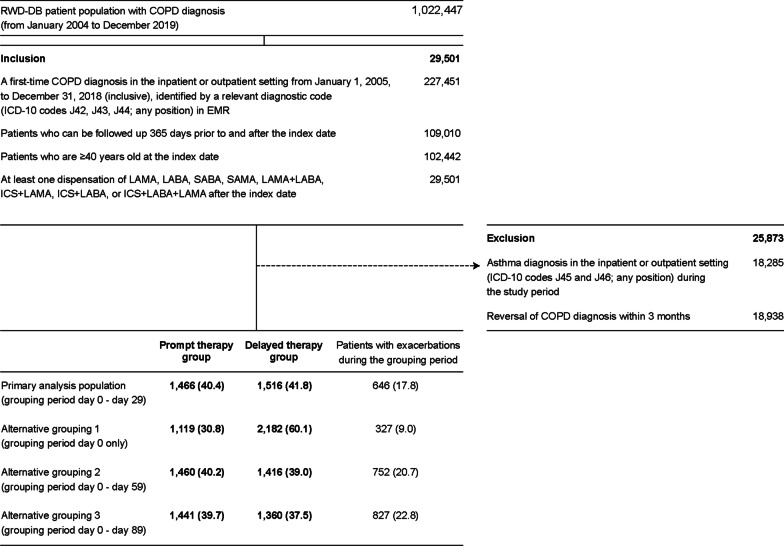
Analysis population. All values are n or n (%). *COPD* chronic obstructive pulmonary disease, *EMR* electronic medical record, *ICD-10* International Statistical Classification of Diseases and Related Health Problems, 10th Revision, *ICS* inhaled corticosteroid, *LABA* long-acting β_2_-agonist, *LAMA* long-acting muscarinic antagonist, *RWD-DB* Real World Data database, *SABA* short-acting β_2_-agonist, *SAMA* short-acting muscarinic antagonist

A total of 1,022,447 patients diagnosed with COPD between January 2004 and December 2019 were identified from the RWD database (Fig. 2). Of the patients who met the inclusion criteria (n = 29,501), those diagnosed with asthma in the inpatient or outpatient setting (n = 18,285) and those with a reversal of COPD diagnosis within 3 months (n = 18,938) were excluded. Thus, a total of 3628 patients were included for further analysis: prompt therapy group (1466 [40.4%]), delayed therapy group (1516 [41.8%]), and patients who experienced exacerbations during the grouping period (within the first 30 days of diagnosis; 646 [17.8%]).

The mean age of patients in the prompt therapy group was 73.4 years, with 15.1% (n = 222) of patients aged 40–64 years and 84.9% (n = 1244) aged ≥ 65 years. The mean age of patients in the delayed therapy group was 72.6 years, with 17.5% (n = 265) of patients aged 40–64 years and 82.5% (n = 1251) aged ≥ 65 years. The proportion of men was higher in both groups (prompt 83.3% [n = 1221]; delayed 82.8% [n = 1256]). Differences in the distribution of baseline covariates are summarized using the standardized mean difference (SMD; Table 1). The SMD was > 0.1 for smoking status, lung function test history, systemic corticosteroid use, antibiotic prescriptions, phenotype, comorbidities, physician’s medical specialty, and facility size. The frequency of lung function tests was higher in the prompt therapy group (prompt 45.3%; delayed 12.7%). CB (prompt 12.6%; delayed 37.7%) and unclassified (code J44) (prompt 52.8%; delayed 25.3%) were more prevalent in the prompt therapy group. The Charlson Comorbidity Index (moderate) (prompt 3.4%; delayed 1.7%) and the rates of lung cancer, malignant neoplasm of bladder, and lower respiratory tract infection were higher in the prompt therapy group. The number of facilities with ≥ 500 beds was higher in the prompt therapy group (prompt 65.0%; delayed 44.6%).

**Table 1:** Baseline characteristics

Characteristic	Prompt therapy group	Delayed therapy group	Standardized difference
Number of patients, N	1466	1516	
Age, n (%)			
40–64 years	222 (15.1)	265 (17.5)	0.06
≥ 65 years	1244 (84.9)	1251 (82.5)	
Missing	0 (0.0)	0 (0.0)	
Mean, years	73.4	72.6	0.08
Sex, n (%)			
Male	1221 (83.3)	1256 (82.8)	0.01
Female	245 (16.7)	260 (17.2)	0.01
Missing	0 (0.0)	0 (0.0)	–
Smoking status, n (%)			
Current smoker	97 (6.6)	48 (3.2)	0.16
Non-smoker	0 (0.0)	0 (0.0)	–
Ex-smoker	10 (0.7)	0 (0.0)	0.12
Unknown	1359 (92.7)	1468 (96.8)	0.19
Body mass index at and 365 days prior to the index date (kg/m^**2**^)			
Available, n (%)	145 (9.9)	86 (5.7)	
Mean	22.132	22.081	0.01
Missing, n (%)	1321 (90.1)	1430 (94.3)	
Lung function test, n (%)			
Present	664 (45.3)	193 (12.7)	0.77
Not present	802 (54.7)	1323 (87.3)	
Medication			
Systemic corticosteroid, n (%)			
Used	56 (3.8)	29 (1.9)	0.11
Not used	1410 (96.2)	1487 (98.1)	
Number of systemic corticosteroids			
Available, n (%)	1466 (100.0)	1516 (100.0)	
Mean	0.2	0.1	0.05
Antibiotics prescription, n (%)			
Used	391 (26.7)	309 (20.4)	0.15
Not used	1075 (73.3)	1207 (79.6)	
Number of antibiotic prescriptions			
Available, n (%)	1466 (100.0)	1516 (100.0)	
Mean	1.4	1.2	0.05
Comorbidity			
Charlson Comorbidity Index			
Mild, n (%)	1339 (91.3)	1432 (94.5)	0.12
Moderate, n (%)	50 (3.4)	26 (1.7)	0.11
Severe, n (%)	77 (5.3)	58 (3.8)	0.07
Available, n (%)	1466 (100.0)	1516 (100.0)	
Mean	1.2	0.9	0.15
Missing, n (%)	0 (0.0)	0 (0.0)	
Number of patients in hospital owing to non-COPD exacerbations or acute respiratory failure			
Available, n (%)	1466 (100.0)	1516 (100.0)	
Mean	0.3	0.2	0.14
Missing, n (%)	0 (0.0)	0 (0.0)	
Lung cancer, n (%)			
Yes	151 (10.3)	91 (6.0)	0.16
No	1315 (89.7)	1425 (94.0)	
Malignant neoplasm of the bladder, n (%)			
Yes	16 (1.1)	4 (0.3)	0.10
No	1450 (98.9)	1512 (99.7)	
Lower respiratory tract infections, n (%)			
Yes	61 (4.2)	123 (8.1)	0.17
No	1405 (95.8)	1393 (91.9)	
Facility information, n (%)			
< 20 beds	8 (0.5)	4 (0.3)	0.04
20 to < 100 beds	7 (0.5)	15 (1.0)	0.06
100 to < 300 beds	211 (14.4)	363 (23.9)	0.24
300 to < 500 beds	287 (19.6)	458 (30.2)	0.25
≥ 500 beds	953 (65.0)	676 (44.6)	0.42
Missing	0 (0.0)	0 (0.0)	–
Phenotype, n (%)			
Chronic bronchitis	185 (12.6)	572 (37.7)	0.60
Emphysema	671 (45.8)	652 (43.0)	0.06
Unclassified	774 (52.8)	384 (25.3)	0.59
Specialty information, n (%)			
Pulmonology, respiratory medicine	719 (49.0)	471 (31.1)	0.37
Other	747 (51.0)	1045 (68.9)	0.37

*COPD* chronic obstructive pulmonary disease

Mean time to treatment initiation was 2.9 days (median 0.0 days) and 45.9 months (median 35.0 months) in the prompt and delayed therapy groups, respectively (Additional file 2: Fig. S1 and Additional file 3: Fig. S2). Approximately 70% of patients initiated inhaled therapy on the day of COPD diagnosis (day 0) in the prompt therapy group, whereas more than 20% of patients initiated inhaled therapy after 60 months in the delayed therapy group. LAMA monotherapy was the most common initial therapy in both groups (prompt 61.9%; delayed 38.2%), and LAMA + LABA combination use was similar between the groups (prompt 17.2%; delayed 17.9%; Additional file 4: Fig. S3).

In the delayed therapy group, approximately 50% of patients had received long-acting bronchodilators and/or short-acting bronchodilators before the first exacerbation (Additional file 1: Table S3).

### Time to first exacerbation and AERs in the overall population

**Fig. 3:**
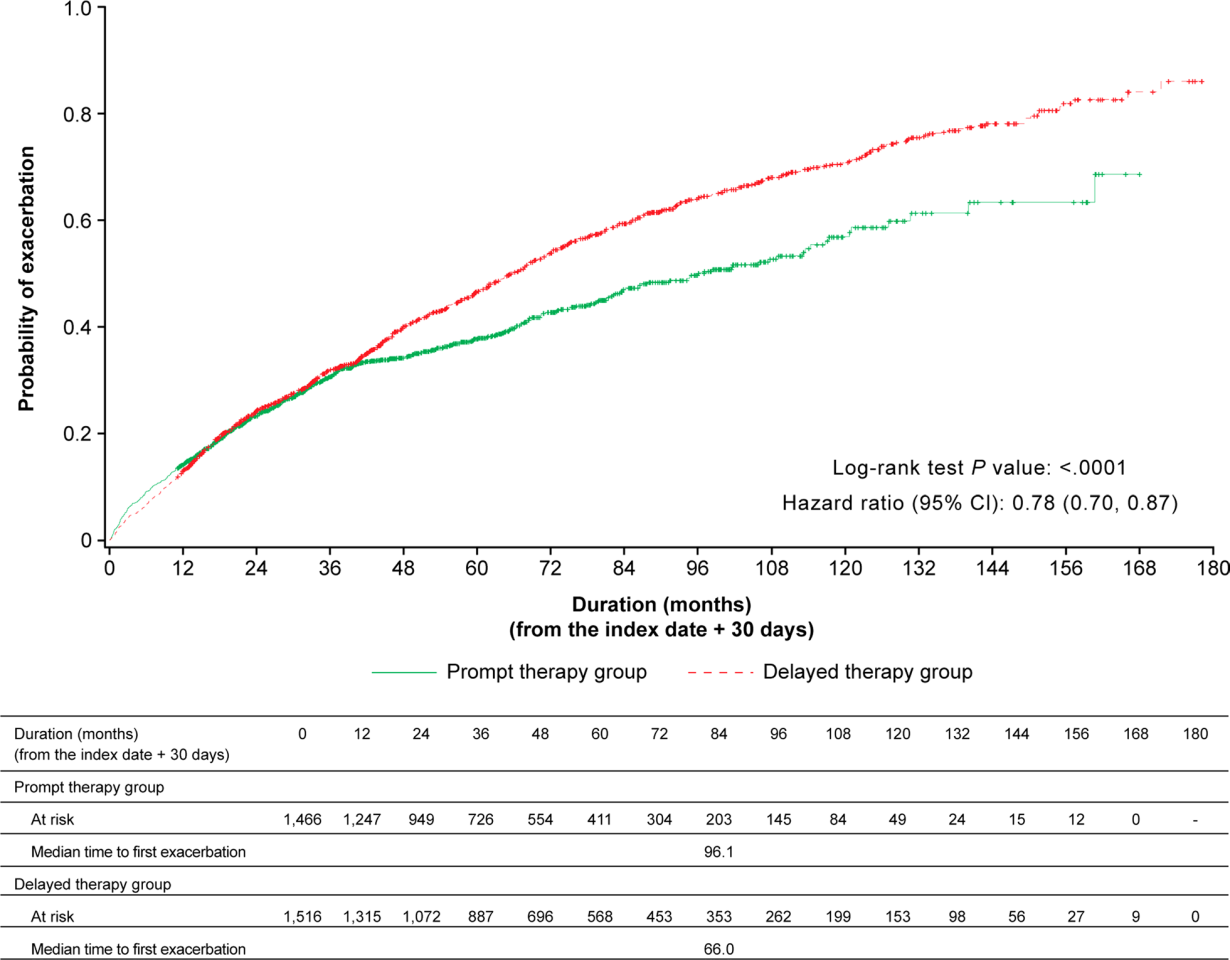
Moderate or severe exacerbation in the prompt and delayed therapy populations. *CI* confidence interval

Time to first exacerbation was longer in the prompt therapy group than in the delayed therapy group (Fig. 3). The median time to first exacerbation was 96.1 months in the prompt therapy group vs 66.0 months in the delayed therapy group (unadjusted HR 0.78 [95% CI 0.70, 0.87]; adjusted HR 0.72 [95% CI 0.65, 0.81]; Table 2).

**Table 2:** Hazard ratios for exacerbations

	Exacerbation (n/N)		Hazard ratio (95% CI)^a^	
	Prompt therapy group	Delayed therapy group	Unadjusted	Adjusted
Full analysis set	37.7 (552/1466)	58.4 (885/1516)	0.78 (0.70, 0.87)	0.72 (0.65, 0.81)
By phenotype				
Chronic bronchitis	40.5 (75/185)	64.2 (367/572)	0.72 (0.56, 0.92)	0.70 (0.54, 0.90)
Emphysema	38.7 (260/671)	56.6 (369/652)	0.81 (0.69, 0.96)	0.80 (0.68, 0.94)
Unclassified	37.3 (289/774)	53.6 (206/384)	0.91 (0.76, 1.09)	0.82 (0.68, 0.99)

*COPD* chronic obstructive pulmonary disease, *CI* confidence interval

^a^Calculated by Cox proportional hazards model

Covariates in pre-index period include age, sex, number of comorbidities, use of systemic corticosteroids, use of antibiotics, and number of patients in hospital owing to non-COPD exacerbations or acute respiratory failure

Unadjusted and adjusted RRs for the prompt therapy group compared with the delayed therapy group were 0.74 (95% CI [0.65, 0.84]) and 0.71 (95% CI [0.62, 0.80]), respectively (Table 3).

**Table 3:** Annual moderate or severe exacerbation rate ratios

	Treatment initiation group (n)	Follow-up period (person-years)	Number of exacerbations	Rate (95% CI)^a^	Rate ratio (95% CI)^a^	
					Unadjusted	Adjusted
Full analysis set	Delayed (1516)	9491.1	2418	0.30 (0.28, 0.33)	Reference	Reference
	Prompt (1466)	6822	1287	0.22 (0.20, 0.25)	0.74 (0.65, 0.84)	0.71 (0.62, 0.80)
By phenotype						
Chronic bronchitis	Delayed (572)	3631.7	1065	0.33 (0.30, 0.37)	Reference	Reference
	Prompt (185)	900.9	192	0.26 (0.21, 0.33)	0.78 (0.60, 1.02)	0.75 (0.58, 0.79)
Emphysema	Delayed (652)	4273.7	986	0.29 (0.25, 0.33)	Reference	Reference
	Prompt (671)	3505.9	577	0.20 (0.17, 0.23)	0.69 (0.57, 0.84)	0.69 (0.57, 0.83)
Unclassified	Delayed (384)	2206.8	507	0.27 (0.23, 0.33)	Reference	Reference
	Prompt (774)	3199.6	710	0.25 (0.22, 0.29)	0.92 (0.73, 1.15)	– (–, –)

*COPD* chronic obstructive pulmonary disease, *CI* confidence interval

^a^Calculated by Cox proportional hazards model

Covariates in pre-index period include age, sex, number of comorbidities, use of systemic corticosteroids, use of antibiotics, and number of patients in hospital owing to non-COPD exacerbations or acute respiratory failure

### Time to first exacerbation and AERs by phenotype

In the CB and emphysema phenotype subgroups, similar to the results of the overall population, the time to first exacerbation was longer in the prompt therapy group than in the delayed therapy group (unadjusted HRs for CB and emphysema 0.72 [95% CI 0.56, 0.92] and 0.81 [95% CI 0.69, 0.96], respectively; Table 2). The adjusted HRs for the CB and emphysema phenotypes were consistent with the unadjusted HRs. Unadjusted and adjusted HRs for the prompt therapy group in the unclassified subgroup were 0.91 (95% CI [0.76, 1.09]) and 0.82 (95% CI [0.68, 0.99]), respectively (Table 2).

Unadjusted RRs for the annual estimated rates of moderate or severe exacerbations for CB, emphysema, and unclassified were 0.78 (95% CI [0.60, 1.02]), 0.69 (95% CI [0.57, 0.84]), and 0.92 (95% CI [0.73, 1.15]), respectively (Table 3). The annual rates of moderate or severe exacerbation were higher in the CB group than in the overall population (prompt 0.26; delayed 0.33).

### Time to first exacerbation by time periods

**Fig. 4:**
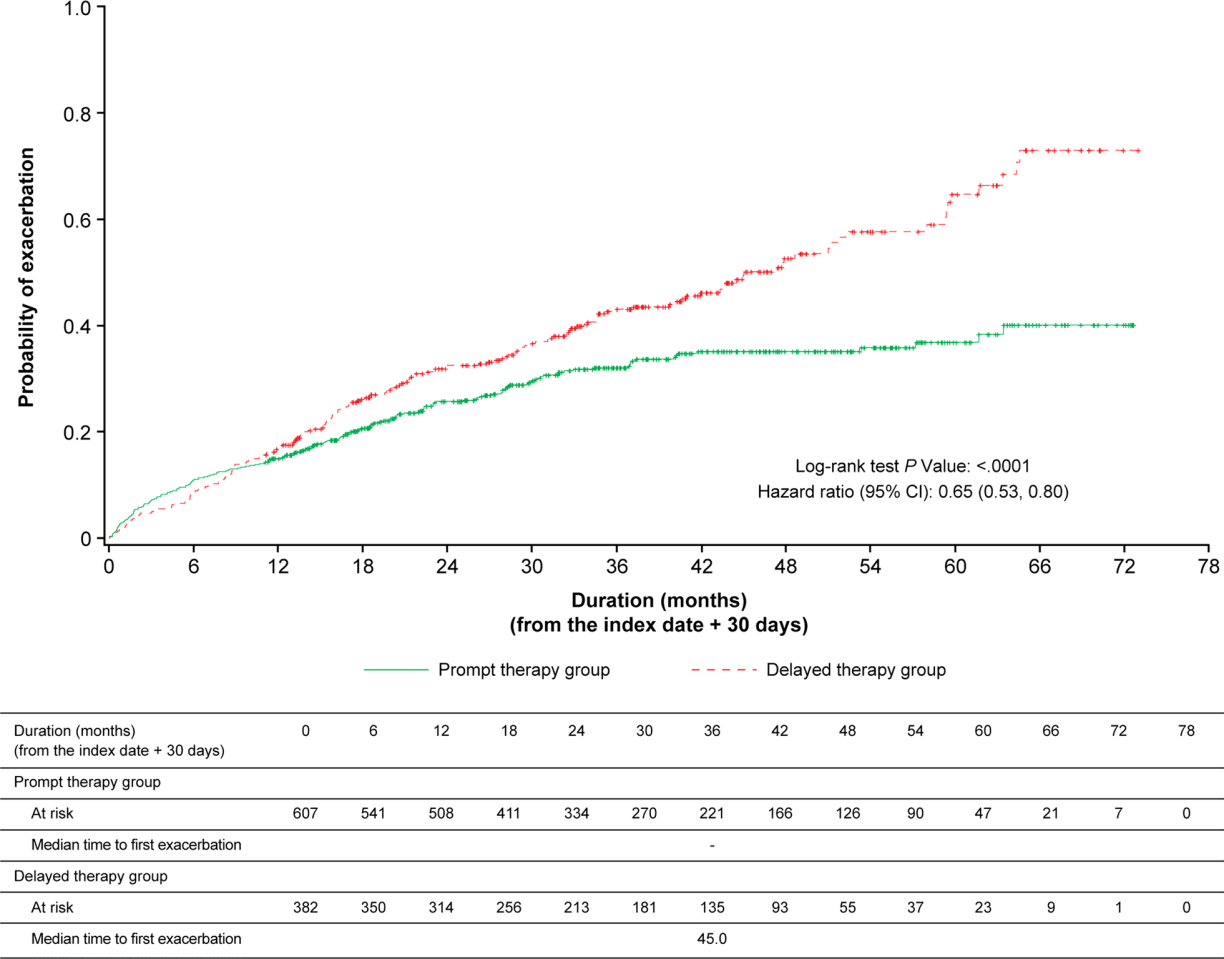
Moderate or severe exacerbation in the prompt and delayed therapy populations with index date after November 1, 2013 (LAMA + LABA product launch). *CI* confidence interval, *LABA* long-acting β_2_-agonist, *LAMA* long-acting muscarinic antagonist

Time to first exacerbation in patients with an index date after the launch of LAMA + LABA in Japan (after November 1, 2013) was increased in the prompt therapy group, with an unadjusted HR of 0.65 (95% CI [0.53, 0.80]) (Fig. 4). Moreover, in the subgroup with an index date after LAMA + LABA recommendation according to COPD guidelines in Japan, the unadjusted HR for the prompt therapy group compared with the delayed therapy group was 0.56 (95% CI [0.23, 1.39]) (Additional file 5: Fig. S4 and Table 4). Among patients with an index date after the launch of LAMA + LABA, the proportion of patients who initiated LAMA monotherapy (38.4%) and LAMA + LABA (34.3%) was similar in the prompt therapy group. In the delayed therapy group also, the proportion was similar between LAMA monotherapy (24.9%) and LAMA + LABA (27.5%), while the proportion of patients receiving no treatment was 30.1% (Additional file 6: Fig. S5).

**Table 4:** Hazard ratios for exacerbations in subgroups stratified by the index date

	Exacerbation (n/N)		Hazard ratio (95% CI)^a^	
	Prompt therapy group	Delayed therapy group	Unadjusted	Adjusted
Index date after November 1, 2013	30.5 (185/607)	46.3 (177/382)	0.65 (0.53, 0.80)	0.64 (0.52, 0.79)
Index date after April 1, 2018	17.6 (12/68)	27.6 (8/29)	0.56 (0.23, 1.39)	0.55 (0.21, 1.42)

*COPD* chronic obstructive pulmonary disease, *CI* confidence interval

^a^Calculated by Cox proportional hazards model

Covariates in pre-index period include age, sex, number of comorbidities, use of systemic corticosteroids, use of antibiotics, and number of patients in hospital owing to non-COPD exacerbations or acute respiratory failure

### Time to first exacerbation by grouping periods

As patients who initiated inhaled therapy within 30 days of diagnosis had a prolonged time to first exacerbation, we evaluated whether these results may have been driven by the selected time periods. Therefore, we expanded our analysis to include longer time windows. The results of the analysis with different grouping periods were similar to those seen with the 30-day grouping period. The time to first exacerbation was longer in the prompt therapy group than in the delayed therapy group (HR 0.79 [95% CI 0.71, 0.88] for the grouping period of 0 days, 0.77 [95% CI 0.69, 0.86] for the grouping period of 60 days, and 0.75 [95% CI 0.67, 0.84] for the grouping period of 90 days) (Additional file 1: Table S4).

## Discussion

We assessed real-world evidence to elucidate the benefit of early initiation of inhaled long-acting bronchodilators. Patients with COPD who initiated long-acting bronchodilators within 30 days of diagnosis had a prolonged time to first exacerbation and decreased AER compared with those with delayed initiation of therapy. Prompt therapy similarly prolonged the time to first exacerbation in patients with CB and emphysema. Among patients stratified by the index date following the launch of LAMA + LABA therapy in Japan, the time to first exacerbation was prolonged in the prompt therapy group after diagnosis. Our findings indicate a beneficial effect of prompt therapy, and thus lend credence to the recommendation that patients initiate timely inhaled long-acting bronchodilator treatment upon COPD diagnosis.

The use of long-acting anticholinergic bronchodilator therapies in patients with mild and moderate COPD can reduce the risk and severity of exacerbations compared with placebo [17, 19]. Prompt diagnosis in patients with COPD is associated with a longer time to first exacerbation, as well as a decreased exacerbation rate [20, 21], and prompt initiation of triple therapy after two moderate exacerbations or one severe exacerbation is associated with decreased morbidity and economic burden [22]. The findings in the current study are consistent with these previous observations. As exacerbations can irreversibly reduce lung function [23] and accelerate disease progression [24], prompt initiation of bronchodilator therapy may further contribute to better disease outcomes by reducing exacerbations.

The baseline sociodemographic characteristics of the patients included in the present cohort were consistent with those in previous studies [25, 26], although the prevalence of each comorbidity was lower than that described previously in the Japanese population [27]. Since the current database only includes registered facilities, information regarding comorbidities may not be available for patients if they were diagnosed or received treatment at non-registered facilities. Although the SMD was > 0.1 for age, systemic corticosteroid use, antibiotic prescription, Charlson Comorbidity Index, lung cancer, and lower respiratory tract infection, the overall SMD suggests adequate matching between the prompt therapy group and the delayed therapy group. The prevalence of emphysema was similar between the current study and a previous study (36–44%) [28]. In the delayed therapy group, some patients initiated long-acting bronchodilators following an episode of the first exacerbation, suggesting that a lack of obvious clinical signs could delay treatment initiation in COPD. Lung function testing was more commonly performed in the prompt therapy group (45.3%) than in the delayed therapy group (12.7%). Moreover, COPD diagnosis was more commonly made by pulmonologists in the prompt therapy group (49.0%) than in the delayed therapy group (31.1%). Healthcare professionals rely on spirometry to confirm a diagnosis of COPD [29]; therefore, the timing of therapy initiation may be affected by the specialty of the treating physician. The rates of moderate or severe exacerbations among patients analyzed in our cohort were consistent with those described previously in the Japanese population [25, 26, 30]. The incidence of COPD exacerbations may be lower in Japan than in other countries because Japanese physicians often use asthma-COPD overlap as a disease label, resulting in an underreporting of the incidence of COPD exacerbations [30].

Initiation of inhaled bronchodilators within 30 days of diagnosis was associated with suppression of future exacerbations in CB. The relatively higher annual rates of moderate or severe exacerbations among patients with CB were consistent with the results of a previous study that described an association between bronchial wall thickness and increased AER [10]. The time to first exacerbation was also prolonged in the prompt therapy group in the emphysema subgroup. There was a tendency toward reduction in exacerbations, with an unadjusted HR of 0.91 and an unadjusted RR of 0.92, in the unclassified group. In line with a previous study suggesting an increased risk of exacerbations with CB or emphysema [10], our findings indicate the benefit of timely initiation of inhaled therapy for patients with COPD irrespective of disease phenotype.

The prolonged time to first exacerbation was more clearly observed approximately 36 months after diagnosis in the overall population. Unintentional exclusion of high-risk patients may have minimized the difference between the two groups up to 36 months as patients who experienced exacerbations within 30 days of the index date were excluded from the study. Interestingly, the time to first exacerbation was also prolonged in selected patients with the index date after the launch of LAMA + LABA therapy, with a clear prolongation of approximately 12 months after diagnosis. A greater proportion of patients in the prompt therapy group initiated LAMA + LABA therapy in the subgroup with an index date after the launch of LAMA + LABA (Additional file 6: Fig. S5). LAMA + LABA combinations are more effective than the individual components because of their distinct mechanisms [31–33]. Use of LAMA + LABA may have been responsible for the difference in the prolonged time to first exacerbation between the overall population and those with an index date after LAMA + LABA launch.

The definition of “prompt” was assessed by comparing the time to first exacerbation between the prompt therapy and delayed therapy groups stratified by different grouping periods (0 days, 60 days, and 90 days). The time to first exacerbation was prolonged in the prompt therapy group in all three subgroups. Similar results between those classified by different grouping periods further support the beneficial effect of early initiation of inhaled therapy following COPD diagnosis. There was a proportionally small difference in prompt therapy patients between the grouping periods of 0 days and 90 days; thus, further studies are required to determine the most appropriate timing of prompt treatment initiation.

Some limitations of this study should also be acknowledged. First, there are no clear guidelines for the optimal timing to initiate inhaled therapy for COPD following diagnosis. Although the current study defined “within 30 days of diagnosis (including the day of diagnosis)” as “prompt” based on expert opinion, evidence-based guidance on the right timing for patients with COPD to initiate therapy requires further evaluation. The time to first exacerbation was prolonged in the prompt therapy group, including the alternative grouping period subgroups, thereby confirming the robustness of the results of the 30-day grouping period. Second, in the current study, only facilities registered in the RWD database were analyzed. Thus, the AERs may have been underestimated if the enrolled patients had received treatment in non-registered facilities. Third, the algorithm for identifying moderate or severe exacerbations was not validated in this study and exacerbation was defined based on previous literature [34]; however, the AERs in this study were consistent with those reported previously. Fourth, the effect of disease severity was not considered in the analysis because data on forced expiratory volume in 1 s were not available in the RWD database. The frequency of exacerbations tends to be higher at more advanced stages of the disease, with recurrence of exacerbations observed even in milder COPD [35]. Finally, ICS use in combination with long-acting bronchodilators may provide a positive clinical effect [36]. The current study did not consider the role of ICS, and the impact of ICS on time to first exacerbation needs to be investigated further.

## Conclusions

This is the first study to demonstrate an association between early initiation of bronchodilators and favorable clinical outcomes in patients with COPD. Initiation of bronchodilator therapy within 30 days of diagnosis positively affected the clinical outcomes in patients with CB or emphysema. Timing of initiation of inhaled bronchodilator therapy is a critical factor for future exacerbations; hence, patients should be advised to initiate bronchodilators in a timely manner.

## Supplementary Information

**Additional file 1: Table S1.** ICD-10 codes. ICD-10, International Statistical Classification of Diseases and Related Health Problems, 10th Revision. **Table S2.** ATC classification codes. ATC, Anatomical Therapeutic Chemical; ICS, inhaled corticosteroid; LABA, long-acting β_2_-agonist; LAMA, long-acting muscarinic antagonist; SABA, short-acting β_2_-agonist; SAMA, short-acting muscarinic antagonist. **Table S3.** Proportion of delayed therapy patients who used long-/short-acting bronchodilators before exacerbation. **Table S4.** Hazard ratios for exacerbations in subgroups stratified by periods of grouping. COPD, chronic obstructive pulmonary disease; CI, confidence interval. ^a^Calculated by Cox proportional hazards model. Covariates in pre-index period include age, sex, number of comorbidities, use of systemic corticosteroids, use of antibiotics, and number of patients in hospital owing to non-COPD exacerbations or acute respiratory failure.

**Additional file 2: Figure S1.** Distribution of time between diagnosis and inhaler therapy initiation in the prompt therapy group

**Additional file 3: Figure S2.** Distribution of time between diagnosis and inhaler therapy initiation in the delayed therapy group

**Additional file 4: Figure S3.** Class-wise distribution of initial therapy in the prompt and delayed therapy groups. ICS, inhaled corticosteroid; LABA, long-acting β_2_-agonist; LAMA, long-acting muscarinic antagonist.

**Additional file 5: Figure S4.** Moderate or severe exacerbations in the prompt and delayed therapy populations with index dates after April 1, 2018. CI, confidence interval.

**Additional file 6: Figure S5.** Class-wise distribution of initial therapy in the prompt therapy and delayed therapy populations with index dates after November 1, 2013 (LAMA + LABA product launch). ICS, inhaled corticosteroid; LABA, long-acting β_2_-agonist; LAMA, long-acting muscarinic antagonist.

## Data Availability

The analyses were conducted on medical records data provided under a commercial license, which the authors are unable to share.

## Abbreviations

AER: Annual exacerbation rate
CB: Chronic bronchitis
CI: Confidence interval
COPD: Chronic obstructive pulmonary disease
EMR: Electronic medical record
HR: Hazard ratio
ICD-10: International Statistical Classification of Diseases and Related Health Problems, 10th Revision
ICS: Inhaled corticosteroid
LABA: Long-acting β_2_-agonist
LAMA: Long-acting muscarinic antagonist
RR: Rate ratio
RWD: Real World Data
SMD: Standardized mean difference
