# Correction to: Effect of timing of bronchodilator therapy initiation on exacerbations in patients with chronic obstructive pulmonary disease: a retrospective cohort study

**DOI:** 10.1186/s12931-022-02219-y

**Published:** 2022-11-07

**Authors:** Hideyasu Yamada, Isao Matsumoto, Naoyuki Makita, Yoshifumi Arita, Nobuya Hayashi, Kurena Mitsuoka, Naoki Tashiro, Nobuyuki Hizawa

**Affiliations:** 1 Department of Pulmonary Medicine, University of Tsukuba, 1‑1‑1 Tennodai, Tsukuba, Ibaraki 305‑8575 Japan; 2 AstraZeneca K.K., Osaka, Japan

## Correction to: Respiratory Research (2022) 23:255 https://doi.org/10.1186/s12931-022-02184-6

Following publication of the original article [1], the authors identified errors in the mention of the baseline characteristics in Table 1 and covariates in the pre-index period in the Statistical analysis subsection of the Methods section and in the footnotes of Tables 2, 3, 4, and Additional file 1: Table S4. The analysis method for the rate ratios was also incorrectly mentioned as Cox proportional hazards model instead of negative binomial model in the footnotes of Table 3. An instance of duplicated text in the Author contributions section and some minor errors in the Reference section were also noticed.

The original article has been corrected.

1. Statistical analysis subsection under the Methods section:
  Original: Adjusted HRs and RRs and corresponding 95% CIs between groups were also estimated including the treatment initiation group and covariates in the pre-index period (age, sex, number of comorbidities, systemic corticosteroid use, antibiotic use, and inpatient with a COPD exacerbation or acute respiratory failure).
  Corrected to: Adjusted HRs and RRs and corresponding 95% CIs between groups were also estimated including the treatment initiation group and covariates in the pre-index period (age, sex, number of comorbidities, systemic corticosteroid use, antibiotic use, and number of hospitalizations excluding hospitalization due to COPD exacerbation or acute respiratory failure).
2. Table 1:
  Original: Number of patients in hospital owing to non-COPD exacerbations or acute respiratory failure
  Corrected to: Number of hospitalizations excluding hospitalization due to COPD exacerbation or acute respiratory failure
3. Footnotes of Tables 2, 3, 4, and Additional file 1: Table S4:
  Original: Covariates in pre-index period include age, sex, number of comorbidities, use of systemic corticosteroids, use of antibiotics, and number of patients in hospital owing to non-COPD exacerbations or acute respiratory failure
  Corrected to: Covariates in pre-index period include age, sex, number of comorbidities, use of systemic corticosteroids, use of antibiotics, and number of hospitalizations excluding hospitalization due to COPD exacerbation or acute respiratory failure
  Additional file 1 has been replaced with a new file reflecting the revisions made to Table S4.
4. Table 3 footnote:
  Original: ^a^Calculated by Cox proportional hazards model
  Corrected to: ^a^Calculated by negative binomial model
5. Author contributions:
  The last sentence in the Author contributions section of the original article, “All authors read and approved the final manuscript.” has been deleted as this text is already a part of the previous sentence.
6. Reference 7:
  A period has been added before the journal name.
  Original: Paggiaro PL, Dahle R, Bakran I, Frith L, Hollingworth K, Efthimiou J. Multicentre randomised placebo-controlled trial of inhaled fluticasone propionate in patients with chronic obstructive pulmonary disease. International COPD Study Group Lancet. 1998;351(9105):773–80.
  Corrected to: Paggiaro PL, Dahle R, Bakran I, Frith L, Hollingworth K, Efthimiou J. Multicentre randomised placebo-controlled trial of inhaled fluticasone propionate in patients with chronic obstructive pulmonary disease. International COPD Study Group. Lancet. 1998;351(9105):773–80.
7. Reference 9:
  “et al” has been added to the end of the author list.
  Original: Miravitlles M, Soler-Cataluña JJ, Calle M, Molina J, Almagro P, Quintano JA, Spanish guidelines for management of chronic obstructive pulmonary disease (GesEPOC) 2017, et al. Pharmacological treatment of stable phase. Arch Bronconeumol. 2017;53(6):324–35.
  Corrected to: Miravitlles M, Soler-Cataluña JJ, Calle M, Molina J, Almagro P, Quintano JA, et al. Spanish guidelines for management of chronic obstructive pulmonary disease (GesEPOC) 2017. Pharmacological treatment of stable phase. Arch Bronconeumol. 2017;53(6):324–35.
8. Reference 12:
  “et al” has been added to the end of the author list.
  Original: Celli BR, Locantore N, Tal-Singer R, Riley J, Miller B, Vestbo J, et al; ECLIPSE Study Investigators. Emphysema and extrapulmonary tissue loss in COPD: a multi-organ loss of tissue phenotype. Eur Respir J. 2018;51(2):1702146.
  Corrected to: Celli BR, Locantore N, Tal-Singer R, Riley J, Miller B, Vestbo J, ECLIPSE Study Investigators, et al. Emphysema and extrapulmonary tissue loss in COPD: a multi-organ loss of tissue phenotype. Eur Respir J. 2018;51(2):1702146.
9. Reference 22:
  “prompt” in the article title has been revised to title case as “Prompt”
  Original: Tkacz J, Evans KA, Touchette DR, Portillo E, Strange C, Staresinic A, et al. PRIMUS—prompt Initiation of Maintenance Therapy in the US: a real-world analysis of clinical and economic outcomes among patients initiating triple therapy following a COPD exacerbation. Int J Chron Obstruct Pulmon Dis. 2022;17:329–42.
  Corrected to: Tkacz J, Evans KA, Touchette DR, Portillo E, Strange C, Staresinic A, et al. PRIMUS—Prompt Initiation of Maintenance Therapy in the US: a real-world analysis of clinical and economic outcomes among patients initiating triple therapy following a COPD exacerbation. Int J Chron Obstruct Pulmon Dis. 2022;17:329–42.

## Supplementary Information

**Additional file 1: Table S1. **ICD-10 codes. ICD-10, International Statistical Classification of Diseases and Related Health Problems, 10th Revision. **Table S2.** ATC classification codes. ATC, Anatomical Therapeutic Chemical; ICS, inhaled corticosteroid; LABA, long-acting β_2_-agonist; LAMA, longacting muscarinic antagonist; SABA, short-acting β_2_-agonist; SAMA, shortacting muscarinic antagonist. **Table S3.** Proportion of delayed therapy patients who used long-/short-acting bronchodilators before exacerbation. **Table S4.** Hazard ratios for exacerbations in subgroups stratified by periods of grouping. COPD, chronic obstructive pulmonary disease; CI, confidence interval. ^a^Calculated by Cox proportional hazards model. Covariates in pre-index period include age, sex, number of comorbidities, use of systemic corticosteroids, use of antibiotics, and number of hospitalizations excluding hospitalization due to COPD exacerbation or acute respiratory failure.
