# Supplementary figures and images for: Effect of timing of bronchodilator therapy initiation on exacerbations in patients with chronic obstructive pulmonary disease: a retrospective cohort study
Source: Respir Res. 2022 Sep 19;23:255. doi: 10.1186/s12931-022-02184-6 (PMC9487074; doi:10.1186/s12931-022-02184-6)

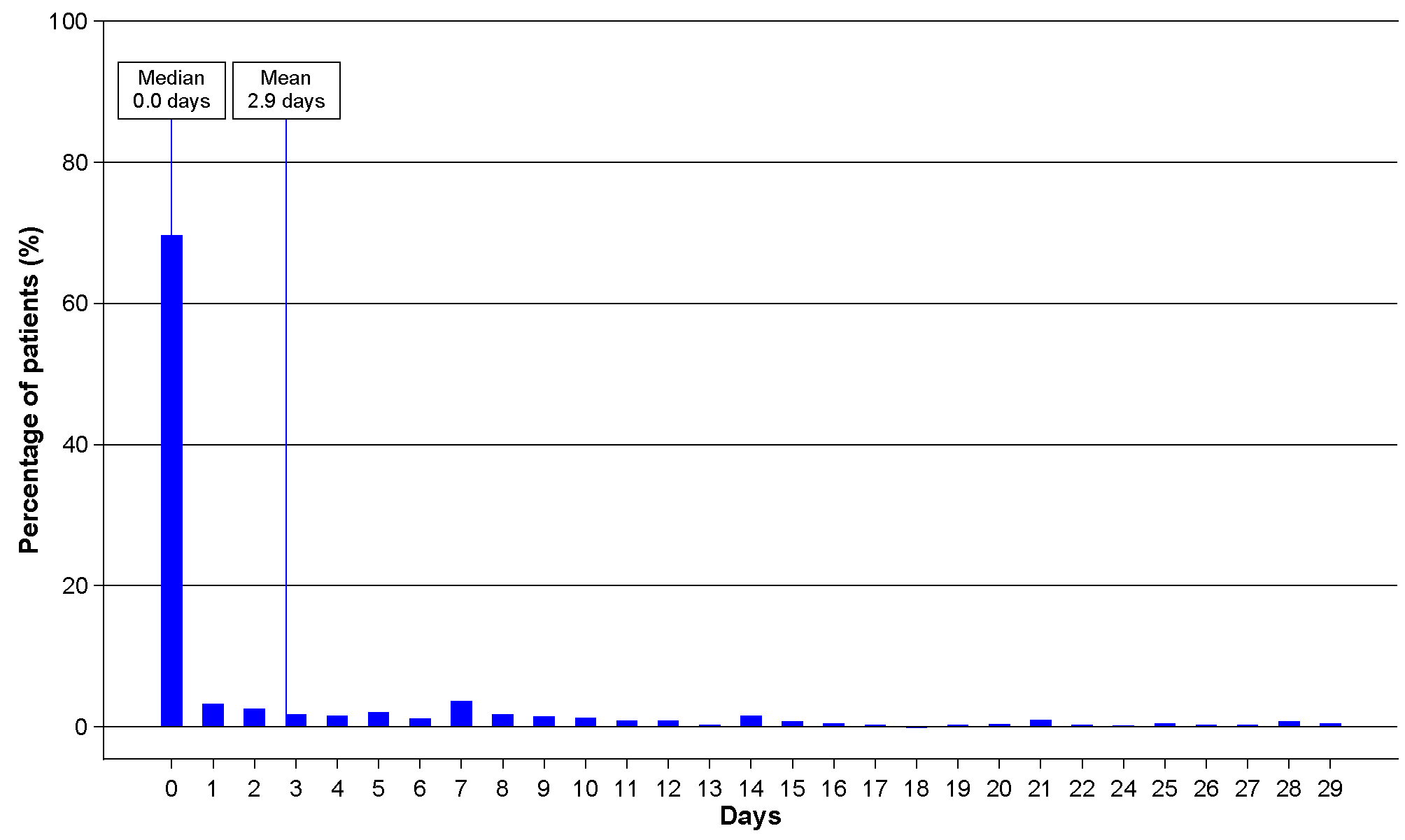

Supplement: Supplementary file 2 — Additional file 2: Figure S1. Distribution of time between diagnosis and inhaler therapy initiation in the prompt therapy group [file 12931_2022_2184_MOESM2_ESM.tif]

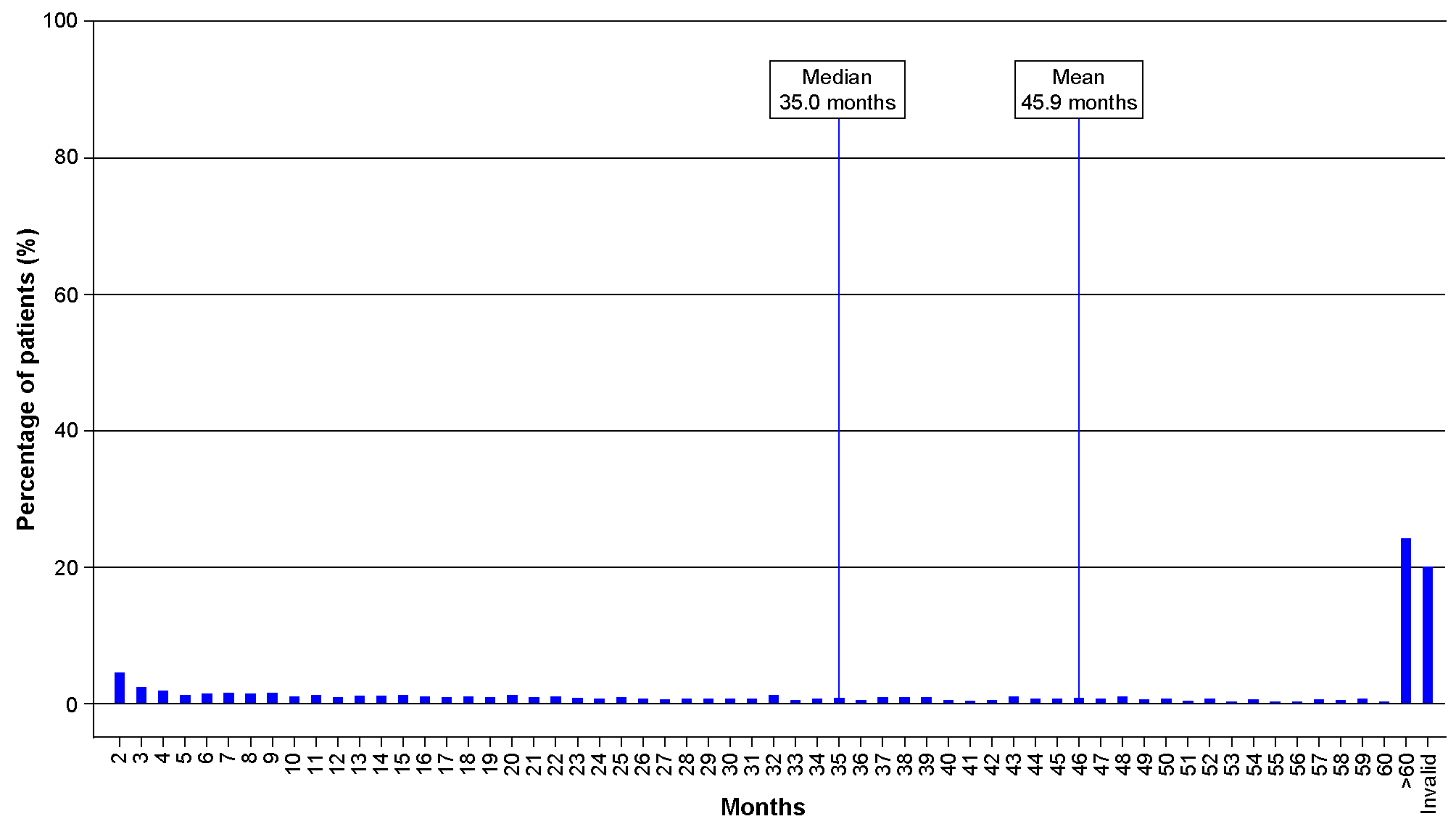

Supplement: Supplementary file 3 — Additional file 3: Figure S2. Distribution of time between diagnosis and inhaler therapy initiation in the delayed therapy group [file 12931_2022_2184_MOESM3_ESM.tif]

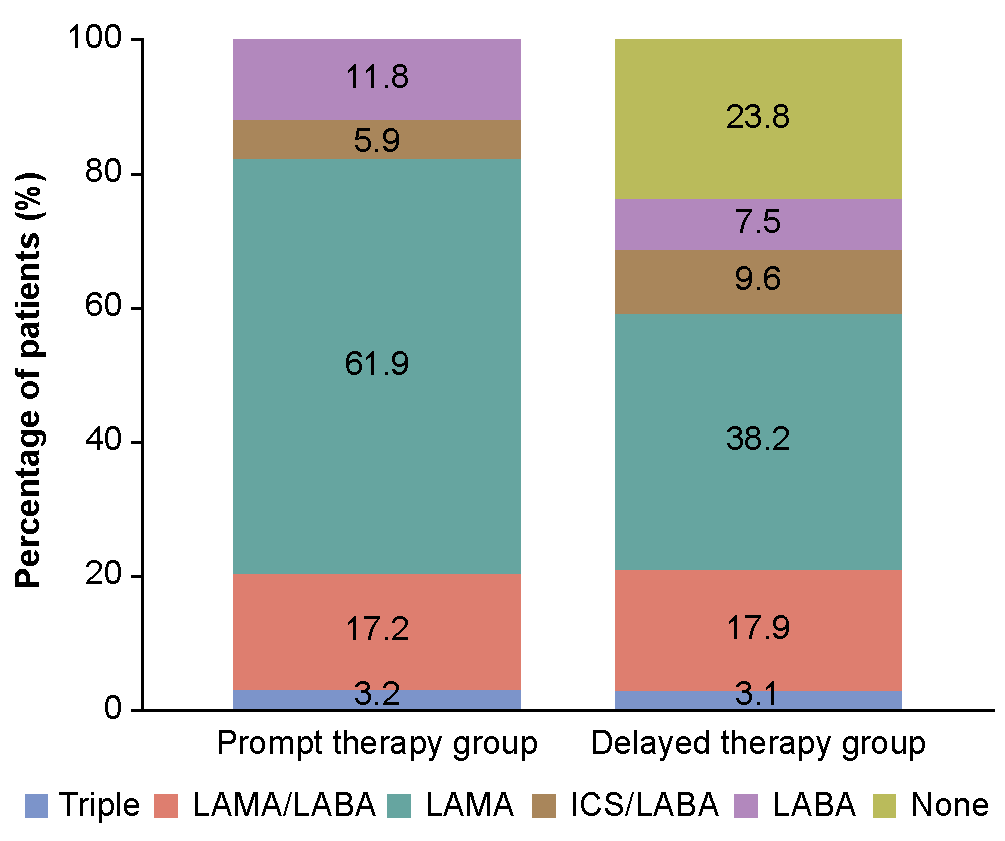

Supplement: Supplementary file 4 — Additional file 4: Figure S3. Class-wise distribution of initial therapy in the prompt and delayed therapy groups. ICS, inhaled corticosteroid; LABA, long-acting β2-agonist; LAMA, long-acting muscarinic antagonist. [file 12931_2022_2184_MOESM4_ESM.tif]

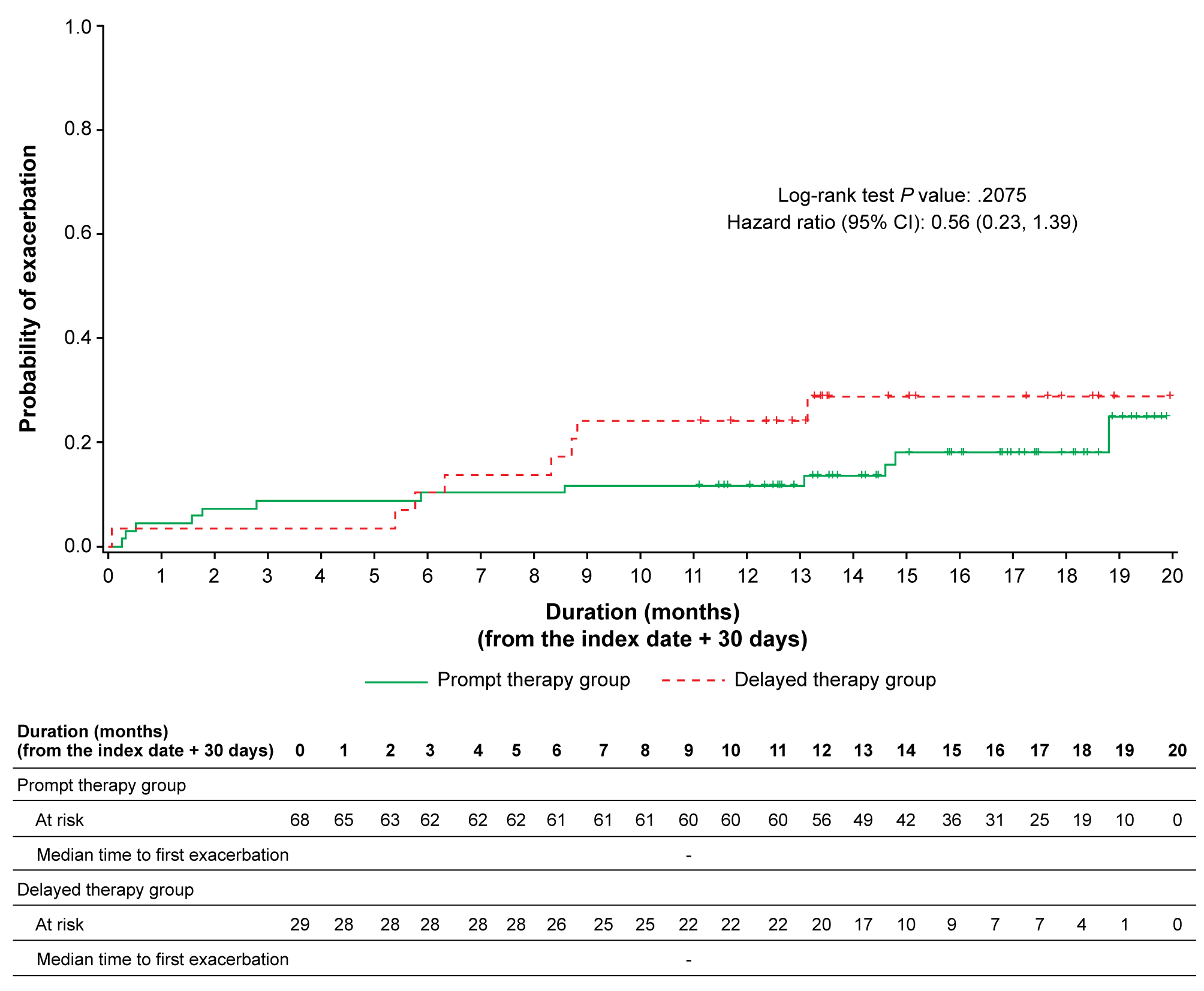

Supplement: Supplementary file 5 — Additional file 5: Figure S4. Moderate or severe exacerbations in the prompt and delayed therapy populations with index dates after April 1, 2018. CI, confidence interval. [file 12931_2022_2184_MOESM5_ESM.tif]

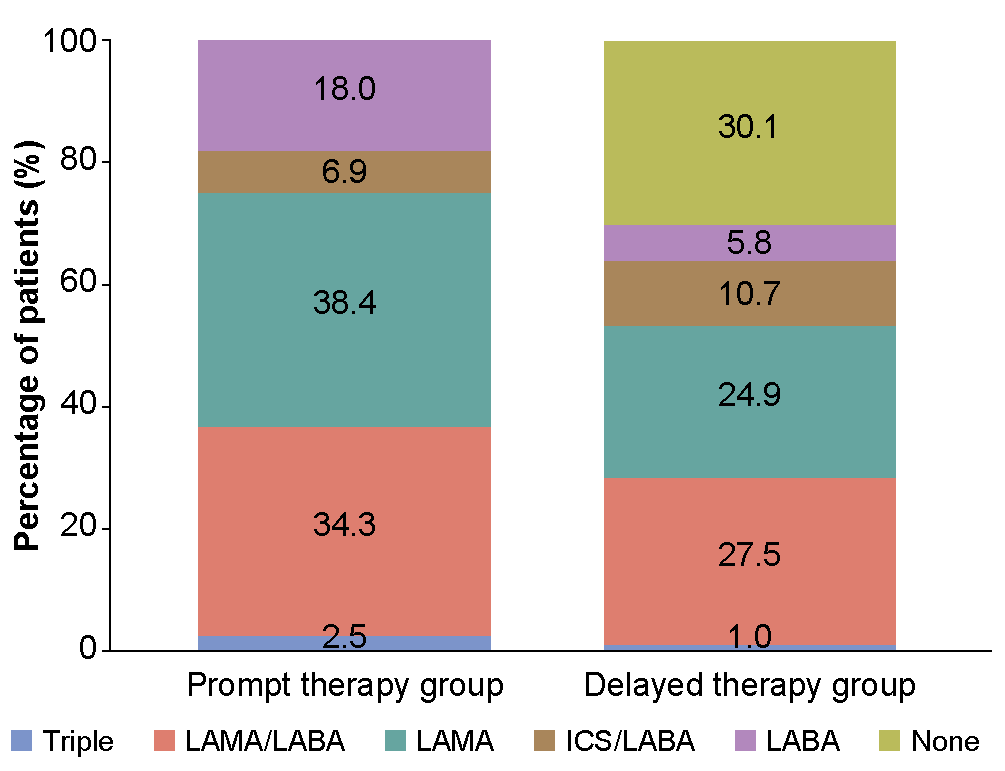

Supplement: Supplementary file 6 — Additional file 6: Figure S5. Class-wise distribution of initial therapy in the prompt therapy and delayed therapy populations with index dates after November 1, 2013 (LAMA + LABA product launch). ICS, inhaled corticosteroid; LABA, long-acting β2-agonist; LAMA, long-acting muscarinic antagonist. [file 12931_2022_2184_MOESM6_ESM.tif]
