# Supplementary material for: Correction to: Effect of timing of bronchodilator therapy initiation on exacerbations in patients with chronic obstructive pulmonary disease: a retrospective cohort study
Source: Respir Res. 2022 Nov 7;23:304. doi: 10.1186/s12931-022-02219-y (PMC9641768; doi:10.1186/s12931-022-02219-y)
Supplement: Supplementary file 1 — Additional file 1: Table S1. ICD-10 codes. ICD-10, International Statistical Classification of Diseases and Related Health Problems, 10th Revision. Table S2. ATC classification codes. ATC, Anatomical Therapeutic Chemical; ICS, inhaled corticosteroid; LABA, long-acting β2-agonist; LAMA, longacting muscarinic antagonist; SABA, short-acting β2-agonist; SAMA, shortacting muscarinic antagonist. Table S3. Proportion of delayed therapy patients who used long-/short-acting bronchodilators before exacerbation. Table S4. Hazard ratios for exacerbations in subgroups stratified by periods of grouping. COPD, chronic obstructive pulmonary disease; CI, confidence interval. aCalculated by Cox proportional hazards model. Covariates in pre-index period include age, sex, number of comorbidities, use of systemic corticosteroids, use of antibiotics, and number of hospitalizations excluding hospitalization due to COPD exacerbation or acute respiratory failure. [file 12931_2022_2219_MOESM1_ESM.docx]

**Additional file 1**

**Table S1** ICD-10 codes

| ICD-10 code: category | ICD-10 code: subcategory | Standard disease name |
| --- | --- | --- |
| A48 | A48.1 | Legionnaires disease |
| B01 | B01.2 | Varicella pneumonia |
| B05 | B05.2 | Measles complicated by pneumonia |
| B25 |  | Cytomegaloviral disease |
| C15 |  | Malignant neoplasm of esophagus |
| C25 |  | Malignant neoplasm of pancreas |
| C34 |  | Lung cancer |
| C50 |  | Malignant neoplasm of breast |
| C67 |  | Malignant neoplasm of bladder |
| F32 | F32.9 | Depressive episodes, unspecified |
| F41 | F41.9 | Anxiety disorders, unspecified |
| I09 |  | Other rheumatic heart diseases |
| I11 |  | Hypertensive heart disease |
| I13 |  | Hypertensive heart and renal disease |
| I21 |  | Acute myocardial infarction |
| I22 |  | Subsequent myocardial infarction |
| I23 |  | Certain current complications following acute myocardial infraction |
| I24 |  | Other acute ischemic heart diseases |
| I24 | I24.9 | Acute ischemic heart disease, unspecified |
| I25 |  | Chronic ischemic heart disease |
| I42 |  | Cardiomyopathy |
| I43 |  | Cardiomyopathy in disease classified elsewhere |
| I48 | I48.9 | Atrial fibrillation and atrial flutter, unspecified |
| I50 |  | Heart failure |
| I63 |  | Cerebral infarction |
| I64 |  | Stroke, not specified as hemorrhage or infarction |
| J10 |  | Influenza due to identified seasonal influenza virus |
| J11 |  | Influenza, virus not identified |
| J13 |  | Pneumonia due to Streptococcus pneumoniae |
| J14 |  | Pneumonia due to Hemophilus influenzae |
| J15 |  | Pneumonia due to Klebsiella pneumoniae |
| J16 |  | Pneumonia due to other infectious organisms, not elsewhere classified |
| J18 |  | Pneumonia, organism unspecified |
| J47 |  | Bronchiectasis |
| J20 |  | Acute bronchitis |
| J21 |  | Acute bronchiolitis |
| J22 |  | Unspecified acute lower respiratory infection |
| J42 |  | Unspecified chronic bronchitis |
| J43 |  | Emphysema |
| J44 | J44.0 | Chronic obstructive pulmonary disease with acute lower respiratory infection |
| J44 | J44.1 | Chronic obstructive pulmonary disease with acute exacerbation, unspecified |
| J44 | J44.8 | Other specified chronic obstructive pulmonary disease |
| J44 | J44.9 | Chronic obstructive pulmonary disease, unspecified |
| J45 |  | Asthma |
| J46 |  | Status asthmaticus |
| J68 |  | Bronchitis and pneumonitis due to chemicals, gases, fumes, and vapors |
| J80 |  | Adult respiratory distress syndrome |
| J85 | J85.1 | Abscess of lung with pneumonia |
| J96 |  | Respiratory failure, not elsewhere classified |
| K21 |  | Gastro-esophageal reflux disease |
| K29 |  | Gastritis and duodenitis |
| P29 |  | Cardiovascular disorders originating in the perinatal period |
| R09 | R09.2 | Respiratory arrest |
| R54 |  | Senility |

ICD-10, International Statistical Classification of Diseases and Related Health Problems, 10th Revision.

**Table S2** ATC classification codes

| ATC code | Class of medication |
| --- | --- |
| R03D1 | ICS |
| R03A3 | LABA |
| R03K2 | LAMA |
| R03A4 | SABA |
| R03K1 | SAMA |
|  | ICS+LAMA |
| R03F1 | ICS+LABA |
| R03L2 | LABA+LAMA |
| R03L3 | ICS+LABA+LAMA |
| R03B | Methylxanthine |
| R03B2 | Slow-release theophylline |

ATC, Anatomical Therapeutic Chemical; ICS, inhaled corticosteroid; LABA, long-acting β_2_-agonist; LAMA, long‑acting muscarinic antagonist; SABA, short-acting β_2_-agonist; SAMA, short-acting muscarinic antagonist.

**Table S3** Proportion of delayed therapy patients who used long-/short-acting bronchodilators before exacerbation

| Number of patients with exacerbation, n | 885 |
| --- | --- |
| History of medication before exacerbation, n (%) |  |
| Used long-acting bronchodilators only | 294 (33.2) |
| Used short-acting bronchodilators only | 75 (8.5) |
| Used both | 38 (4.3) |
| Did not use any | 478 (54.0) |

**Table S4** Hazard ratios for exacerbations in subgroups stratified by periods of grouping

| Grouping period | Exacerbation (n/N) | | Hazard ratio (95% CI)^a^ | |
| --- | --- | --- | --- | --- |
|  | **Prompt therapy**  **group** | **Delayed therapy group** | **Unadjusted** | **Adjusted** |
| 0 days | 38.8  (434/1,119) | 54.8  (1,196/2,182) | 0.79  (0.71, 0.88) | 0.74  (0.66, 0.83) |
| 60 days | 36.2  (528/1,460) | 58.1  (823/1,416) | 0.77  (0.69, 0.86) | 0.72  (0.64, 0.80) |
| 90 days | 34.7  (500/1,441) | 57.7  (785/1,360) | 0.75  (0.67, 0.84) | 0.70  (0.62, 0.78) |

COPD, chronic obstructive pulmonary disease; CI, confidence interval.

^a^Calculated by Cox proportional hazards model.

Covariates in pre-index period include age, sex, number of comorbidities, use of systemic corticosteroids, use of antibiotics, and number of hospitalizations excluding hospitalization due to COPD exacerbation or acute respiratory failure.
